# Osteocyte Cell-Cell Communication Within and Beyond Bone

**DOI:** 10.1007/s11914-026-00964-3

**Published:** 2026-04-10

**Authors:** Astrid D. Bakker, Victor J.B. van Santen

**Affiliations:** Department of Oral Cell Biology, Academic Centre for Dentistry Amsterdam, University of Amsterdam and Vrije Universiteit Amsterdam, Amsterdam, The Netherlands

**Keywords:** Osteocyte communication, Bone remodeling, Signaling molecules, Endocrine signaling, Bone-organ crosstalk, Kidney

## Abstract

**Purpose of Review:**

Osteocytes form an extensive cellular network and communicate both with neighboring bone cells and with distant organs. Here, we review evidence on which tissues osteocytes communicate with, and the molecular “language” they use.

**Recent Findings:**

Recent work has expanded osteocyte communication beyond the classical triad of receptor activator of nuclear factor κB ligand, sclerostin, and fibroblast growth factor 23. Within bone, osteocytes coordinate remodeling through direct cell-cell contacts and rapidly released mediators, acting on timescales relevant to mechanotransduction. Beyond bone, osteocytes signal to adipose tissue, muscle, kidney, cartilage, and vasculature. Extracellular vesicles are increasingly implicated as messengers, as their cargo (RNAs and proteins) can influence stem cell lineage allocation and bone–cartilage interactions. Osteocytes may also support vascular function via mitochondria transfer.

**Summary:**

Osteocytes integrate mechanical and metabolic cues and transmit information through a broader repertoire of signaling molecules than traditionally appreciated. Recognizing the diversity of target cells and mediators will refine concepts of osteocyte-bone and osteocyte-organ crosstalk and highlight mechanisms that could be targeted to reduce skeletal fragility.

## Introduction

Osteoporosis is a major global health issue characterized by reduced bone mass and increased fracture risk [1]. Osteoporosis has long been linked to endocrine changes e.g. after menopause, resulting in an imbalance between bone resorption and bone formation [2]. However, skeletal fragility cannot be understood solely in terms of osteoblast and osteoclast activity. Nowadays, bone is recognized as an active endocrine organ, and osteocytes, which are the most abundant and long-lived cells in mineralized bone, have emerged as orchestrators of skeletal remodeling, but also of systemic mineral homeostasis [3, 4].

Osteocytes form a highly interconnected syncytium of cells that reside within the lacuno-canalicular network inside the bone matrix, from where osteocytes integrate mechanical, metabolic, and inflammatory cues, and translate them into coordinated responses. Osteocytes communicate with neighboring osteoblast-lineage cells, osteoclasts, vascular cells and marrow-derived cells through direct cell-cell contacts and through the production of a diverse repertoire of secreted factors, and potentially communicate with multiple extra-skeletal organs as well, via endocrine-like signals. This communication is clinically relevant because hallmark features of osteoporosis, e.g. increased resorption, impaired formation, compromised bone structure, and reduced resistance against mechanical loads, are shaped by osteocyte-derived signals, and by how these signals propagate through cellular networks within and beyond bone.

Despite the important role of osteocytes in maintaining bone homeostasis, osteocyte communication is frequently discussed quite narrowly in terms of a few canonical osteocyte-derived proteins such as receptor activator of nuclear factor κB ligand (RANKL) and sclerostin. Importantly, the discovery of these crucial factors did reshape bone biology and directly enabled highly effective osteoporosis therapies, underscoring the translational value of defining osteocyte communication pathways. At the same time, an exclusive focus on a small set of molecules, or even only at proteins only, risks overlooking additional mediators, particularly rapidly acting small molecules, lipid derivatives, reactive species, and extracellular vesicle (EV) cargo that may represent complementary targets for preventing or treating skeletal fragility. Indeed, emerging work suggests that osteocytes transmit information through a broader “language” than proteins. In addition, osteocyte signals may extend beyond bone to influence other tissues, as has been extensively described for the osteocyte-kidney axis. Therefore, the purpose of this review is to (i) summarize the recent evidence for osteocyte cell-cell communication with skeletal and extra-skeletal targets, and (ii) describe the major signaling modes that mediate this cross-talk.

### Osteocyte Cell–Cell Communication Within Bone

In this review we will use “osteocyte cell-cell communication” to encompass signaling among osteocytes as well as osteocyte communication to neighboring cells and distant organs. Within bone, osteocytes influence virtually every cell type participating in skeletal remodeling, including their neighboring osteocytes [5–8]. Osteocytes reside within the lacuno-canalicular system and extend dendritic processes through canaliculi, where they form direct contacts with neighboring osteocytes [9], enabling rapid signal exchange across a functional syncytium. The prevailing fluid-flow hypothesis posits that mechanical loading induces interstitial fluid flow within canaliculi, and thereby generates shear stress on osteocyte membranes, which in turn initiates mechanotransduction and downstream signaling.

Fluid shear is a highly potent trigger: It evokes intracellular calcium transients within seconds, and promotes the production and/or release of mediators that regulate osteoblast, osteocyte and osteoclast behavior, including nitric oxide (NO), prostaglandins, and adenosine triphosphate (ATP) [8, 10, 11] (Table 1). In contrast, unloading, immobilization, or microgravity shift osteocyte signaling toward a more catabolic state, characterized by increased expression of the osteoblast inhibitor sclerostin and increased osteoclastogenic output [6, 12]. Microdamage and osteocyte apoptosis represent additional triggers that recruit osteoclasts to targeted remodeling sites. This occurs in part through release of apoptotic bodies and damage-associated molecular patterns (DAMPs), which serve as chemotactic and pro-osteoclastogenic signals [13–15]. Metabolic stressors such as hypoxia, oxidative stress, glucocorticoids, and advanced glycation end products further alter osteocyte viability and signaling output, shifting communication from adaptive remodeling toward bone loss [12, 16].

An additional determinant of osteocyte function is the integrity of the osteocyte network and its surrounding matrix microenvironment. Dentin matrix protein 1 (DMP1), a highly expressed osteocyte matrix protein, is essential for osteocyte maturation and mineral homeostasis, and DMP1 deficiency causes rickets/osteomalacia [17]. Importantly, DMP1 helps maintain lacuno–canalicular structure and osteocyte identity, thereby shaping the conditions under which mechanosensing and perilacunar remodeling occur. In this way, DMP1 can be viewed as part of the “infrastructure” that enables osteocyte communication by stabilizing the cellular and matrix context in which signals are generated and propagated.

Taken together, osteocyte communication links sensing of mechanical stimuli, microdamage, and metabolic cues to coordinated remodeling outputs by osteoblastic cells and osteoclastic cells. Below, we first describe osteocyte-to-osteocyte signaling as the core information-processing network in bone, and then discuss how osteocytes communicate with osteoblast-lineage cells, including their progenitors.

**Table 1:** Osteocyte communication by target tissue / cell type

Target cell/tissue	Signal types	Effect
Osteocytes	Cell-cell via gap junctions (Cx43); Ca²⁺ waves. Release of ATP, PGE₂ via hemichannels. Sema3A production. NO production. [10, 18, 19].	Propagates mechanical-signals, synchronizes survival and transcriptional programs. Affects remodelling balance.
Osteoblasts / lining cells	Anabolic: WNT1, IGF-1, BMP7. Inhibitors: Sclerostin, DKK1, Notum [20–25].	Tunes osteoblastogenesis and bone formation.
Osteoblast progenitors	Tropomyosin1 in EVs WNT1, IGF-1 [15, 26–28].	Promotes osteogenic differentiation; shifts lineage allocation toward osteogenesis.
Osteoclasts/ Osteoclast precursors	RANKL, WNT–TGF‑β axis. Damage signals: HMGB1, DAMPs, apoptotic bodies [5–8, 13, 14, 23, 29].	Induces osteoclastogenesis and targeted resorption after stress/ damage/ disuse.
Immune/myeloid cells in the bone marrow	Chemokines: CCL5, CCL7, CCL3. CSF-1; Gsα pathways; PGRN/ GRN/ NRP1. IL-19 [21, 24, 25, 30–34].	Controls precursor recruitment and myelopoiesis. Supports remodeling / inflammatory responses, e.g. granulopoiesis and neutrophil formation.
Kidney	Endocrine: FGF23 [11].	Regulates phosphate/ Vit D. Contributes to fibrosis/ pathology in injury/CKD [35].
Intestine	Endocrine: FGF23 [36].	Decreases Mg²⁺ absorption; links bone to mineral handling.
Central nervous system	Proteins: FGF23, Sclerostin [37, 38].	Synaptic effects and cognition links; evidence mainly indirect.
Muscle	Protein: WNT3a. EVs (proposed) [15, 39].	Myogenic differentiation (in vitro). In vivo osteocyte-specific evidence limited.
Blood vessels Inside bone	Organelle transfer of mitochondria [40].	Increases angiogenesis and vascular function. Supports transcortical vessel adaptation.
Adipose tissue	Sclerostin, NPY. EVs [15, 41–44].	Enhance adipogenesis. Shifts BMSC fate.
Cartilage	miR-23b-3p, lncRNA: H19, IGF-1 [45–47].	OA progression. Fracture cartilage remodeling.

*ATP *adenosine triphosphate, *BMP7 *bone morphogenetic protein 7, *BMSC* bone marrow stromal cell, *Ca²⁺* calcium ion, *CAV‑1* caveolin‑1, *CCL C‑C* motif chemokine ligand, *CSF‑1* colony‑stimulating factor 1, *Cx43* connexin 43,* DAMPs* damage‑associated molecular patterns,* DKK1* dickkopf‑1, *DMP1* dentin matrix protein 1, *EC* endothelial cells, *EV* extracellular vesicles,* FGF23* fibroblast growth factor 23, *Gsα* stimulatory g protein alpha, *HMGB1* high mobility group box 1, *IGF‑1* insulin‑like growth factor 1,* IL* interleukin, *Mg²⁺* magnesium ion, *miRNA* microRNA, *NO* nitric oxide, *NPY* neuropeptide Y, *OA* osteoarthritis, *OPN* osteopontin, *PGE₂* prostaglandin E₂, *PGRN/GRN *progranulin/granulin, *PTH* parathyroid hormone, *RANKL* receptor activator of nuclear factor κB ligand, S*ema3A* semaphorin 3 A, *TGF‑β* transforming growth factor β, *WNT* wingless‑related integration site protein

### Osteocyte-to-Osteocyte Communication: Mechanotransduction and Network Signaling

Osteocyte-to-osteocyte communication is the most literal interpretation of “osteocyte cell-to-cell communication”, where communication may often happen rapidly, via direct transmission of signals between interconnected cells in the network. At the molecular level, osteocyte network signaling is mediated by gap junctional coupling, largely via connexin 43 (Cx43), enabling transfer of ions and small signaling molecules between osteocytes [11, 12]. Physical dendritic contacts within canaliculi support propagation of calcium waves and other fast signaling events, allowing mechanical information to spread across the network on the timescale of seconds to minutes [8]. Because osteocytes are embedded in mineralized matrix and communicate through a confined lacuno–canalicular space, osteocyte-derived signals are often expected to act locally, i.e. either within the osteocyte network itself or on a limited number of nearby surface cells. This is particularly true for short-lived mediators, such as NO [8], which is produced rapidly in response to mechanical stimulation and diffuses over short distances before being inactivated. Hemichannels provide a regulated route for the release of other short-lived soluble mediators such as ATP and the more stable prostaglandin E₂ (PGE2), which can act on neighboring osteocytes and on cells at the bone surface [11]. Although diffusion through mineralized tissue is limited, mechanically induced canalicular fluid flow may support transport and mixing of soluble factors through canaliculi, thereby facilitating communication between embedded osteocytes and surface-lining cells. In addition to supporting mediator release in response to mechanical cues, Cx43-dependent signaling promotes osteocyte survival and preserves network integrity under stress, linking altered connectivity to osteocyte viability and downstream remodeling outcomes [12].

Besides Cx43-dependent signaling, notch signaling is another typical contact-dependent pathway, in which membrane-bound ligands such as jagged or delta-like proteins activate notch receptors on neighboring cells to induce transcriptional responses. Osteocyte-directed notch activation suppresses bone resorption and increases bone volume, consistent with osteopetrosis-like phenotypes [48, 49]. It is tempting to postulate that direct osteocyte-to-osteocyte communication propagates through notch/jagged communication. However, although notch is a contact-dependent pathway, and osteocytes form an extensive cellular syncytium, current evidence mainly supports a role for osteocyte-intrinsic notch signaling in regulating remodeling, rather than direct osteocyte–osteocyte jagged/notch communication [48, 49]. Further research should elucidate whether there is a role for jagged/notch signaling within the osteocyte network.

While the physiological relevance of EV exchange between osteocytes remains incompletely defined, EVs provide an additional possible route for transferring signals, such as ribonucleic acid (RNA) and protein cargo, locally to other osteocytes and to neighboring cells [15]. Functionally, this layered communication system, i.e. gap junction coupling, hemichannel release of fast mediators, release of de-novo produced proteins, and vesicle-based signaling, allows osteocytes to disseminate mechanical signals and synchronize transcriptional programs such as sclerostin expression to alter recruitment and activity of osteoblasts.

### Osteocyte-to-Osteoblast Communication: Bone Formation and Osteogenic Recruitment

Signaling molecules produced by osteocytes regulate osteoblast activity and bone-lining cell behavior, thereby coupling osteocyte sensing to bone formation. Osteocytes also influence recruitment and differentiation of osteoblast precursors, shaping the osteogenic potential of mesenchymal stromal populations in bone marrow and, potentially, periosteal compartments. Osteocyte-derived morphogens and growth factors, including wingless-related integration site (WNT)1, bone morphogenetic protein 7 (BMP7), and insulin growth factor 1 (IGF-1), support osteoblast differentiation and function and contribute to skeletal adaptation [26–28]. In parallel, osteocytes provide potent inhibitory “brakes” on bone formation. The identification of sclerostin as the product of the SOST gene and its connection to high-bone mass disorders provided a cornerstone for understanding osteocyte-derived inhibition of WNT signaling and bone formation [48, 49]. Canonical WNT inhibitors produced by osteocytes, including sclerostin, dickkopf-1 (DKK1), and the less-well known WNT‑deacylating enzyme notum, exert antagonistic effects that fine-tune osteoblast activity and the formation/resorption balance [23–25]. Osteocyte stress responses can also alter osteocyte–osteoblast coupling. Recent work suggests that osteocyte-derived lipocalin-2 can regulate osteocyte ferroptosis and shift osteocyte-to-osteoblast signaling through WNT-related pathways, with downstream effects on bone formation. If confirmed in vivo, this would provide a direct example of how a defined stress program in osteocytes changes the signals they provide to osteoblasts, linking metabolic stress to impaired bone formation.

A recent in vivo study showed that osteocyte Cx43 hemichannel opening and PGE₂ release are required to direct bone marrow mesenchymal stromal cell commitment, thereby linking mechanical sensing to osteogenic versus adipogenic lineage allocation [20]. This provides one of the clearest mechanistic examples that osteocyte “fast” signaling can control longer-term remodeling outcomes through effects on progenitor fate [20]. In line with this, a recent study identified caveolin-1 (CAV-1) as a regulator of osteocyte communication with osteoclast and osteoblast precursors, affecting recruitment and differentiation of progenitor populations in the marrow environment [21]. This suggests that osteocytes may not only control precursor numbers through secreted factors, but can also modulate how precursor cells interpret those signals [21].

A clear recent development is the finding that osteocyte-derived EVs can directly promote osteoblast differentiation. In 2024, osteocyte EV cargo was shown to include tropomyosin-1 and thereby to enhance osteogenic differentiation and matrix deposition [15]. EV-mediated signaling may be particularly relevant for osteoblast progenitor populations in marrow and periosteum, where vesicle cargo could complement classical morphogens to influence lineage allocation and osteogenic recruitment.

In addition to vesicle-based mediators, matrix-associated molecules such as DMP1 support osteoblast-to-osteocyte maturation and mineral metabolism, whereas osteopontin (OPN) can facilitate migration of mesenchymal progenitors and may support osteoblast recruitment under select conditions [17, 21]. Semaphorin 3 A (Sema3A) has been described as a coupling signal that supports osteoblast activity while restraining osteoclasts, integrating anabolic and antiresorptive actions within a single osteocyte-derived pathway [22]. Together, these pathways position osteocytes not only as regulators of mature osteoblast function, but also as upstream determinants of osteogenic lineage allocation in response to local mechanical and metabolic conditions.

### Osteocyte Cell–Cell Communication Within Bone: Osteoclasts and Bone Marrow

Osteoclastogenesis is tightly controlled by osteocyte-derived signaling molecules that indicate local microdamage, cellular stress, or loss of mechanical stimuli. Osteocytes regulate osteoclast formation and activity through a combination of membrane-bound ligands, secreted cytokines/chemokines, and contact-dependent signaling systems, thereby connecting local sensing of mechanical or damage signals to site-specific bone resorption.

A central osteocyte-derived cue is RANKL. The concept that osteocytes are a dominant physiological source of RANKL in adult bone was established by two back-to-back in vivo studies demonstrating that osteocyte-specific RANKL deletion causes profound defects in osteoclast formation and bone remodeling [17, 21]. More recently, mechanistic work has begun to clarify upstream regulation of osteocyte RANKL under defined microenvironmental conditions. For example, an osteocyte transforming growth factor beta (TGF-β) signaling axis has been proposed to promote osteocyte RANKL production and osteoclastogenesis [29]. In this model, activation of canonical WNT/β-catenin signaling in osteocytes upregulates TGF-β 1/2 expression and increases smad2/3 phosphorylation, thereby activating TGF-β signaling. Smad4 can then bind the RANKL promoter region to enhance RANKL transcription [29]. Osteocyte RANKL production is influenced by local mechanical conditions and by systemic regulators of mineral metabolism such as parathyroid hormone (PTH). Reduced loading, inflammation, and altered calcium-regulating hormone signaling (including PTH/PTH-related protein pathways) can shift osteocytes toward a more osteoclast-supportive program [30].

In the setting of osteocyte injury, necrosis, apoptosis, or senescence, osteocytes release signals that recruit osteoclasts to sites requiring targeted remodeling. High mobility group box 1 (HMGB1) and other DAMPs promote osteoclast recruitment, migration, and differentiation, and osteocyte apoptotic bodies can convey osteoclastogenic activity and initiate localized bone destruction [13, 14, 23]. Recent work further suggests that osteocyte senescence represents a distinct trigger for pro-resorptive signaling: Irradiation-induced senescent osteocytes can over-secrete c–c motif chemokine ligand 3 (CCL3), which has been implicated in remodeling imbalance and may contribute to osteoclast recruitment [24]. These DAMPs signals appear to initiate recruitment, while cytokines and chemokines further amplify osteoclastogenesis in inflammatory or mechanically perturbed environments. Interleukin-6 (IL-6) and c–x–c motif chemokine ligand 10 (CXCL10) can directly stimulate osteoclast formation [25, 50, 51]. IL-6 secretion is triggered by perturbation of primary cilia and by parathyroid hormone receptor signaling [25].

Osteocyte control of osteoclastogenesis is further refined by contact-dependent signaling systems. Importantly, RANKL itself is predominantly presented as a membrane-tethered ligand on osteocytes, and direct cell contact can therefore enhance its osteoclastogenic potency compared with soluble RANKL. Furthermore, ephrin-eph receptor interactions, including eph a2-ephrin a2 and eph b4-ephrinb2, can either enhance or suppress osteoclast differentiation depending on the ligand-receptor pairing and cellular context [52]. These pathways illustrate that osteocyte communication extends beyond soluble mediators to include membrane-tethered ligand-receptor systems that tune osteoclast number and activity.

Beyond effects on osteoclast differentiation at remodeling sites, osteocytes also regulate the bone marrow microenvironment and the availability of osteoclast precursors. Chemokines such as c–c motif chemokine ligand 5 and 7 (CCL5 and CCL7) influence migration of pre-osteoclasts, monocytes, and other myeloid precursors [21, 25], and mechanically regulated CCL7 secretion has been described as a protective osteocyte-derived signal [31]. Osteocytes also provide survival and differentiation signals that shape myeloid lineage output. In particular, osteocyte-derived colony-stimulating factor 1 (CSF-1) is essential for maintaining balanced trabecular remodeling and influences osteoclast progenitor behavior, in part by suppressing oxidative stress pathways [32]. Osteocytes secrete IL-19 which stimulates granulopoiesis and neutrophil formation [34]. In addition, osteocyte signaling through stimulatory g-protein alpha (Gsα)-dependent pathways controls myeloid proliferation and differentiation in bone marrow and peripheral hematopoietic sites [30, 33], and recent studies propose that osteocytes may also regulate osteoclast precursor dynamics through mediators such as progranulin/granulin (PGRN/GRN) and associated co-receptor pathways including neuropilin-1 (NRP1) [30]. Together, these findings show that osteocytes help control immune and hematopoietic cell populations that influence bone remodeling and inflammation.

### Osteocyte Cell-Cell Communication Beyond Bone: Systemic Targets and Endocrine Signals

While endocrine signaling to the kidney via fibroblast growth factor 23 (FGF23) is well established, growing evidence suggests that osteocyte-derived signals may also influence adipose tissue, vasculature, cartilage, and possibly muscle and the central nervous system [11, 15, 39, 53]. Osteocyte endocrine output is regulated not only by local bone cues, but also by systemic challenges. For example, phosphate load, inflammation, iron deficiency, and chronic kidney disease–associated factors all influence FGF23 production, while parathyroid hormone regulates expression of osteocyte-derived inhibitors of bone formation such as sclerostin. Thus, osteocyte signaling reflects an integration of skeletal demands with whole-body homeostasis.

### Kidney and Intestine: Well-Established Classic Endocrine Communication

The kidney remains the most extensively studied osteocyte target organ. FGF23 regulates renal phosphate reabsorption and vitamin D (Vit D) metabolism [11], and this axis is strongly influenced by systemic milieu. FGF23 production is tightly regulated by osteocyte-derived DMP1 and phosphate-regulating endopeptidase homolog x-linked (PHEX), which act as key upstream modulators of FGF23 expression and activity. Disruption of this osteocyte-controlled axis is a hallmark of inherited hypophosphatemic disorders and underscores that osteocytes regulate phosphate handling not only by secreting FGF23, but also by controlling its set-point within mineralized matrix. In injured renal tissue, FGF23 can activate pro-fibrotic pathways [35], illustrating that osteocyte-derived endocrine signals may shift from mineral homeostasis toward maladaptive effects under disease conditions. Clinically, inflammatory cytokines, iron deficiency, phosphate load, and uremic factors can drive disproportionate FGF23 elevations, potentially contributing to cardiovascular pathology in chronic kidney disease. The intestine also responds to osteocyte-derived cues, as systemic FGF23 suppresses transcellular magnesium transport in the duodenum and jejunum, linking osteocyte activity to gastrointestinal mineral handling [36]. In addition, endocrine communication to the reproductive system has been described, as FGF23 influences testicular weight, sperm parameters, and calcium responsiveness in human spermatozoa [54], suggesting a role in male reproductive physiology.

### Cartilage: Well-Supported Local Communication Through Non-Classical Mediators

Besides osteocyte-kidney communication, communication between osteocytes and cartilage has in the recent years become a rather well-established example of bone-organ signaling. Osteocyte-specific TGF-β signaling has recently been shown to be mechanosensitive and required for maintaining coupled homeostasis of articular cartilage and subchondral bone [55]. Osteocytes also regulate cartilage behavior during fracture repair: osteocyte-specific deletion of insulin-like growth factor 1 (IGF-1) alters chondrocyte behavior and unexpectedly accelerates bony union [45], indicating that osteocyte signals can modulate endochondral remodeling processes. Furthermore, osteocyte-derived EVs carrying microRNA (miR)-23b-3p have been shown to promote cartilage degeneration and accelerate osteoarthritis progression [46]. This is a particularly important recent development because it demonstrates that osteocytes can influence cartilage through RNA cargo rather than proteins (such as IGF-1) alone.

Beyond microRNAs, osteocytes are increasingly recognized as a source of regulatory noncoding RNAs that can modify joint outcomes. Long noncoding RNAs (lncRNAs) are RNA transcripts longer than ~ 200 nucleotides that do not encode proteins, but can regulate gene expression by acting as scaffolds for chromatin modifiers, regulating transcription factor access, or by binding microRNAs and preventing them from repressing their target genes. Their importance lies in the fact that they can integrate multiple signaling inputs and exert durable regulatory effects on cell behavior. Recent work targeting the long noncoding RNA H19 in subchondral bone osteocytes showed that modulating this pathway can alleviate cartilage degradation in osteoarthritis [47]. This suggests that osteocyte noncoding RNA signaling may represent a mechanistically novel and potentially druggable route for influencing bone–cartilage crosstalk. Taken together this work positions osteocytes as upstream regulators of joint integrity, linking altered loading to coordinated changes in bone as well as cartilage.

### Adipose Tissue: Emerging Osteocyte-to-Fat Signaling

Several osteocyte-derived molecules act on adipose tissue. Sclerostin promotes adipocyte differentiation in 3T3-L1 cells, induces bone marrow adipogenesis, and increases thermogenic gene expression (uncoupling protein 1; UCP1) in peripheral fat depots [41–43]. Neuropeptide Y (NPY) secreted by osteocytes further enhances adipogenic differentiation of mesenchymal stromal cells [44]. In addition, recent work suggests that osteocyte-derived EVs can influence stromal lineage allocation, consistent with a mechanism by which bone-derived vesicle cargo may contribute to adipogenic versus osteogenic fate decisions [15]. Although these findings are still emerging, they broaden the view of osteocytes as regulators of systemic metabolism.

### Central Nervous System and Muscle: Intriguing, But Evidence Remains Limited

Osteocyte-derived factors may also influence the central nervous system. FGF23 can increase synaptic density in hippocampal neurons, while elevated sclerostin has been linked to impaired synaptic plasticity and memory formation in aging and neurodegenerative contexts [37, 56]. However, for many reported neurocognitive effects, the cell-of-origin remains difficult to establish, because the underlying disease models involve systemic inflammation and broad changes in signal-molecule production across multiple tissues. Therefore, while osteocyte-to-brain communication is biologically plausible, osteocyte-specific perturbation studies are still needed to define its physiological relevance.

Communication of bone with muscle has received substantial attention over the past decades, but the strength of evidence with respect to osteocyte-to-muscle communication varies markedly between studies. Osteocytes definitely do produce factors that have the ability to affect muscle [38], but ability does not mean proven biological relevance. Osteocyte-derived WNT3a has been reported to promote myogenic differentiation in muscle progenitors [39], but again, the field still lacks many direct in vivo demonstrations that osteocyte-specific signals are necessary and sufficient to drive physiologically meaningful muscle adaptations. This does not argue against the existence of a bone-muscle axis, rather it emphasizes that osteocyte-derived contributions remain incompletely defined compared with endocrine pathways such as FGF23–kidney signaling.

### Osteocyte-Derived Organelle-Transfer, Vesicles and RNAs: Expanding the Osteocyte “Language”

Across these extra-skeletal targets, a recurring theme is that osteocyte communication extends beyond classical protein mediators (Table 2). EVs and their cargo, including proteins, microRNAs, and long noncoding RNAs, provide a mechanism for transferring complex regulatory information to recipient cells. Recent studies show that EV cargo can influence osteogenic differentiation and matrix deposition [15], and can mediate bone-to-cartilage and bone-to-stroma interactions [46, 47]. In addition, communication with the vascular compartment has gained strong mechanistic support. Osteocytes can support vascular function through mitochondria transfer to endothelial cells in transcortical vessels, a process that promotes angiogenesis and vascular performance [57]. These newer modes of communication suggest that osteocytes can coordinate skeletal and extra-skeletal responses through several distinct routes, including vesicle cargo and organelle transfer. Future research should assess which of these mechanisms operate in vivo and under which physiological conditions.

**Table 2:** Emerging osteocyte “languages” of osteocyte cell-cell communication

Signalling modality	Example mediator(s)	Target(s) / effect
EV protein cargo	EV-Tropomyosin-1 [15].	Enhanced osteoblast differentiation and matrix deposition; may bias BMSC fate.
EV microRNAs	EV-miR-23b-3p [46].	Bone-to-cartilage signaling; promotes OA progression.
lncRNAs	H19 [47].	Subchondral osteocyte pathways; alleviates cartilage degeneration in OA.
Cell-intrinsic miRNAs	miR-21 [56].	Sex-dependent osteocyte viability and bone mechanical properties.
Organelle transfer	Mitochondria transfer to EC [40].	Enhanced angiogenesis; vascular support (novel non-soluble mechanism).

*EC *endothelial cells, *EV* extracellular vesicles, *lncRNA *long noncoding RNA, *miRNA* microRNA, *OA *osteoarthritis, *EV-Tpm1* extracellular vesicle-associated tropomyosin-1, *BMSC* bone marrow stromal cell, , *H19* long noncoding RNA H19

## Conclusions

Collectively, the literature supports osteocytes as primary orchestrators in skeletal communication, regulating formation and resorption through diverse signaling routes. Osteocytes also communicate beyond bone and kidney, specifically to adipose tissue, muscle, kidney, cartilage, and vasculature. A key recent development is that osteocyte signaling extends beyond classical protein pathways. EVs have emerged as plausible carriers of complex information, with evidence that their cargo (RNAs and proteins) can influence stromal lineage allocation and mediate bone–cartilage interactions. Another striking extension of osteocyte communication is organelle transfer to endothelial cells, suggesting direct osteocyte support of the vascular compartment. Together, these advances broaden the osteocyte “language” and expand the range of target tissues that may be influenced by bone. For osteoporosis, this matters because mechanical unloading, aging, and metabolic stress can shift osteocyte output toward reduced formation and increased resorption, and newly described mediators may provide additional routes for intervention.

## Key References

- Liu N, Ma Y, Gong W, Shao X, Shi T, Li L, et al. Osteocyte-derived extracellular vesicles mediate the bone-to-cartilage crosstalk and promote osteoarthritis progression. Nat Commun. 2025; 16(1).
  - ○ Provides direct evidence that osteocyte-derived extracellular vesicles mediate bone-to-cartilage signaling and can drive osteoarthritis progression, establishing EV microRNA cargo as a functional inter-tissue communication modality.
- Liao P, Chen L, Zhou H, Mei J, Chen Z, Wang B, et al. Osteocyte mitochondria regulate angiogenesis of transcortical vessels. Nat Commun. 2024; 15(1).
  - ○ Demonstrates a non-soluble communication mechanism in which osteocytes transfer mitochondria to endothelial cells to support angiogenesis of transcortical vessels, expanding the osteocyte 'language' to organelle transfer.
- Wang R, Mehrjou B, Dehghan-Banian D, Wang BYH, Li Q, Deng S, et al. Targeting Long Noncoding RNA H19 in Subchondral Bone Osteocytes and the Alleviation of Cartilage Degradation in Osteoarthritis. Arthritis Rheumatol. 2025;77(3):283-97.
  - ○ Shows that long noncoding RNA H19 in subchondral bone osteocytes is mechanistically linked to cartilage degeneration and that targeting this pathway alleviates osteoarthritis features, highlighting IncRNA-mediated bone-cartilage crosstalk.
- Wang ZX, Lin X, Cao J, Liu YW, Luo ZW, Rao SS, et al. Young osteocyte-derived extracellular vesicles facilitate osteogenesis by transferring tropomyosin-1. J Nanobiotechnology. 2024;22(1):1-19.
  - ○ Identifies osteocyte EV protein cargo (tropomyosin-1) as an anabolic signal that enhances osteogenesis and matrix deposition, supporting EV protein transfer as a mechanistic route for osteocyte-to-osteoblast/progenitor communication.Identifies osteocyte EV protein cargo (tropomyosin-1) as an anabolic signalthat enhances osteogenesis and matrix deposition, supporting EV proteintransfer as a mechanistic route for osteocyte-to-osteoblast/progenitorcommunication

## Data Availability

No datasets were generated or analysed during the current study.

## Declarations

### Large Language Models

After completion of a systematic screening of the literature in PubMed by the authors, and manually addition of all the papers that were deemed applicable after in depth review by the authors, ChatGPT was prompted to identify potential key scientific papers that appeared to still have been missed by the authors. The limited number of additional papers retrieved via ChatGPT were included only if deemed appropriate after independent in-depth review of the suggested paper by the authors, following the same strict standards as our main screening. ChatGPT was also used to refine sentence structure, check grammar, and standardize proof‑editing, including abbreviation consistency. No scientific conclusions were drawn by ChatGTP, or by the authors based on output solely from ChatGPT, and the authors take full responsibility for the accuracy and integrity of the content. The AI model was explicitly not used to generate data or references, and no confidential information was entered into the tool.
